# Twelve years of chiari-like malformation and syringomyelia scanning in Cavalier King Charles Spaniels in the Netherlands: Towards a more precise phenotype

**DOI:** 10.1371/journal.pone.0184893

**Published:** 2017-09-21

**Authors:** Katrien Wijnrocx, Leonie W. L. Van Bruggen, Wieteke Eggelmeijer, Erik Noorman, Arnold Jacques, Nadine Buys, Steven Janssens, Paul J. J. Mandigers

**Affiliations:** 1 KU Leuven Department of Biosystems, Livestock Genetics, Leuven, Belgium; 2 University of Utrecht, Department of Clinical Sciences of Companion Animals, Utrecht, The Netherlands; 3 Dierenkliniek den Heuvel, Best, The Netherlands; 4 Cavaliers for Life foundation, Oudenaarde, Belgium; University of Sydney Faculty of Veterinary Science, AUSTRALIA

## Abstract

Chiari-like malformation (CM), syringomyelia (SM) and middle ear effusion (also called PSOM) are three conditions that frequently occur in Cavalier King Charles Spaniels (CKCS). Both CM and SM are currently screened in the Netherlands prior to breeding and are graded according to the British Veterinary Association’s Kennel Club (BVA/KC) scheme. This study evaluated the prevalence and estimated genetic parameter of CM, SM and middle ear effusion from 12 years of screening results. For SM, the classical method using the BVA/KC scheme, was compared with exact measuring of the central canal dilation. For CM, the BVA/KC scheme was compared with a more detailed scheme. Next to this the presence of microchip artifacts was assessed. 1249 screening of 1020 dogs were re-evaluated. Results indicated the presence of CM in all dogs, suggesting it has become a breed-specific characteristic. And although different grades of CM were observed, the condition did not deteriorate over time. SM was present in 39% of the dogs and a clear age effect was demonstrated, with SM increasing with age. This emphasizes the importance of screening at appropriate age, since SM can worsen with increasing age. One alternative is to promote repeated measures. The presence of middle ear effusion in this study was 19%–21% for dogs younger than 3 years, and 32%–38% for dogs older than 3 years. In as much as 60%, microchip artifacts were noticed, leading to the recommendation to place microchips in another location in breeds that are susceptible to developing SM. Finally, this study estimated the heritability of CM in this population, due to the lack of phenotypic variance, to be very low at 0.02–0.03. The heritability for SM central canal dilatation to be 0.30, compared to 0.13 for the classical BVA/KC method, using a model including the age effect and the combined effect of veterinary clinic and year of the evaluation. Genetic correlations were rather small, ranging from 0.16–0.33. As a conclusion, screening for SM and CM in the entire population should be maintained, and a selection scheme against SM should be based on estimated breeding values for the exact measurement of the central canal dilatation.

## Introduction

Chiari malformation (CM) is the herniation of the cerebellum through the foramen magnum [1]. In the Cavalier King Charles Spaniel (CKCS) it has been described as a Chiari-like malformation as it is a combination of two causes: an abnormally small caudal cranial fossa [2], and increased cerebellar volume [3], and is diagnosed using MRI scans. The condition occurs frequently in brachycephalic toy breeds like the CKCS, Griffon Bruxellois, Affenpinscher, Chihuahua and Papillons [1,4]. Although CM is often referred to as the cause of syringomyelia (SM), recent work has proven that there is a relation between the two disorders but one cannot simply conclude its causality [2,5]. Both CM as well as SM have been found to be endemic in the CKCS [6]. SM can present itself as a dilation of the central canal or as fluid-containing cavities or syrinxes within the spinal cord. The latter term is used if the dilation is more than 2 mm [1]. Diagnosis is made by magnetic resonance imaging (MRI) of the spinal cord, where the central canal dilation (CCD) or presence of a syrinx, is assessed. SM is a typical late-onset disease. Dogs with severe MRI findings develop SM as young dogs, but the majority of predisposed dogs gradually develop SM as they grow older [7]. About 35% of the dogs having SM actually display the clinical signs associated with the disease [8].

Both CM and SM are currently graded according to the British Veterinary Association’s Kennel Club (BVA/KC) scheme. This grading system grades CM from 0 (no Chiari-like malformation), 1 (cerebellum indented (not rounded)) to 2 (cerebellum impacted into, or herniated through the foramen magnum). SM is graded as 0 (normal), 1 (central canal dilation or separate syrinx, internal diameter of less than 2 mm) to 2 (syringomyelia present, diameter of more than 2 mm or pre-syrinx with or without central canal dilation). In addition to these grades, age is also included in the SM grading system: a (more than 5 years of age), b (3 to 5 years of age) or c (younger than 3 years of age).

The complex nature of this disorder as well as the availability of both pedigree data and screening results makes it a suitable candidate for the use of estimated breeding values (EBVs) as an alternative to phenotypical selection. EBVs are predictions of the genetic value of an animal, which can be estimated using phenotypic information of the animal and its relatives, while accounting for environmental effects [9]. EBVs have already extensively been used in other livestock species to improve their health and production traits [10]. Also in dogs, EBVs have been calculated for complex inherited diseases, such as for example hip- and elbow dysplasia [11–14]. SM is found to be heritable, and to the knowledge of the authors only two previous studies estimated a heritability, with moderate to high genetic values [15,16].

The aim of this study was to evaluate the prevalence of CM and SM based on MRI scans, and to estimate their heritability in CKCS evaluated during 12 years of screening in Belgium and the Netherlands. Breeders have been scanning as of 2004 and have based their selection on the MRI reports they received. We compare two methods of assessing SM, firstly the current method using the BVA/KC scheme and secondly using the exact width of the central canal dilation (CCD) or syrinx. We also compared two methods of assessing CM, the current BVA/KC scheme, and a more detailed scheme with extra classes for the severely affected animals. We evaluate the possibilities of both methods on selection based on the magnitude the heritability and determine whether CM and SM have declined over time. Additionally, the presence of middle ear effusion (also called primary secretory otitis media or PSOM), was evaluated, as well as whether microchip artifacts were present or not.

## Materials and methods

### MRI scans

MRI scans were obtained from seven different (veterinary) MRI centers located in Belgium (Orion—Herentals), and the Netherlands (Department of Clinical Sciences of Companion Animals, Utrecht University, Dierenkliniek den Heuvel—Best, Veterinair MRI centrum Dordrecht, Dierenkliniek Drachten, MCD Amsterdam, and the Isala Diaconessen hospital of Meppel). Only scans submitted to obtain breeding permission were evaluated. The selected period runs from the beginning of 2004 till the end of 2015. During this time frame all MRI centers used low field MRI scanners of 0.2, 0.3 or 0.4 Tesla.

Additional information obtained included gender, color, and age of the dog at the moment the scan was made. Pedigree data were supplied by the Cavaliers For Life foundation combining information from the Dutch Kennel Club “Raad van Beheer” and the Belgian Kennel Club “Koninklijke Maatschappij Sint-Hubertus”.

### MRI scan evaluation

All scans were made according to the CM/SM protocol published earlier in the BVA/KC scheme. These included transverse and sagittal T1 and T2 weighted images of the head and the spinal cord. All MRI images were re-evaluated using the Osirix DICOM viewer graphical software [17] at the department of Clinical Sciences of Companion Animals, Faculty of Veterinary Medicine, by a Diplomate of the European College of Veterinary Neurology (ECVN; Paul Mandigers) and a Diplomate of the European College of Veterinary Diagnostic Imaging (ECVDI; Leonie van Bruggen). All scans were evaluated according to the BVA/KC scheme (https://www.bva.co.uk/Canine-Health-Schemes/CM-SM-Scheme/) giving detailed information on the existence of CM and SM. Additionally, the scans were evaluated for a number of supplementary features.

The presence of CM was scored into five grades instead of three, of which grade 0 (no Chiari malformation) and 1 (cerebellum indented (not round), cerebrospinal fluid (CSF) still visible between the cerebellum and brainstem) are comparable with the BVA/KC scheme. The BVA/KC scheme grade 2 is defined as cerebellum impacted into or herniated through the opening at the rear of the skull (the foramen magnum). We added to this grade 2, a grade 3 (the hind part of the cerebellum is herniated and tongue shaped) and grade 4 (the hind part of the cerebellum is severely herniated and clearly tongue shaped).

Middle ear effusion, or Primary Secretory Otitis Media (PSOM) was scored for both middle ears, left and right side separately, as 0 = not present, 1 = minimal material visible, 2 = clearly present, and 3 = completely full middle ear. Lastly, the presence of a microchip artifact was scored as 0 = not present, 1 = present but it does not interfere with SM diagnosis, 2 = present and it does interfere with SM diagnosis but the majority of the spinal cord is visible and indicate a SM 1 or 2 grading. If an artifact is visible and does not enable a thorough evaluation of the spinal cord, even if a large part appears to be normal, the grade would be 3. If the scan cannot be evaluated at all—the chip artifact is too extended the grade would again be 3. Hence, the artifact would only be scored 2, if the SM diagnosis would be at least be grade SM 1 or SM 2. This to avoid that an artifact scored 2 would allow a favorable grading whereas it could be differently.

### Genetic parameters for the SM phenotype

For both SM and CM evaluation according to the BVA/KC scheme each score was assigned to a numerical value from 0 to 2. For the newly developed CM classification, a numerical value was assigned from 0–4. In a first step, non-genetic factors that might have an influence on the phenotypes of SM and CM were assessed using generalized linear mixed models implemented in the SAS software (Version 9.4, SAS Institute Inc., Cary, NC 27513–2414, USA). Sex, age at scanning, veterinary clinic taking the scan and owner were considered as fixed factors. Alternatively, veterinary clinic was combined with year of evaluation (VCY) and assessed as random factor, and accounts for the experience of the veterinarian in this way. The Akaike information criterion (AIC) was used as a selection criterion to evaluate the most suitable model [18]. After this preliminary analysis, variance components and heritability estimates were calculated with a single trait linear animal model, including the factors that were identified as significant in above-mentioned explanatory analysis:

$${SM}_{ijk}=\mu +g_{i}+{vcy}_{j}+a_{k}+e_{ijk}$$

$${CM}_{jk}=\mu +{vcy}_{j}+a_{k}+e_{jk}$$

Here *SM*_*ijk*_ is the central canal dilation (CCD), *CM*_*jk*_ is the CM score, μ is the population mean, *g*_*i*_ is the effect of age of the animal at screening as a covariable, *vcy*_*j*_ is the random veterinary clinic*year effect, *a*_*k*_ is the additive genetic effect of the animal, and *e*_*ijk*_ are the residual values.

Variance components were estimated using Misztals’ F90 family of programs, including RENUMF90, a renumbering program and AIREMLF90 [19], which used an animal model. Heritability estimates were computed as the proportion of the phenotypic variance (σ^2^_p_) that is explained by the additive genetic variance (σ^2^_a_). Estimated breeding values were provided by AIREMLF90 as well. To evaluate the effectiveness of the new phenotypes, heritability estimates of the CCD will be compared to the estimates based on the classical BVA/KC scheme which divides SM into three different classes. Both the model with and without the VCY factor were tested and compared to evaluate the possible effect of the experience of the veterinarian For CM the newly developed classification scheme and the BVA/KC scheme were compared as well, but only for the model with VYC. From the best models for each phenotype, estimates for the age factor, VCY and animals were obtained. Genetic correlations between SM and CM were estimated using bivariate analyses on pairs of traits.

## Results

### MRI scans

**Fig 1:**
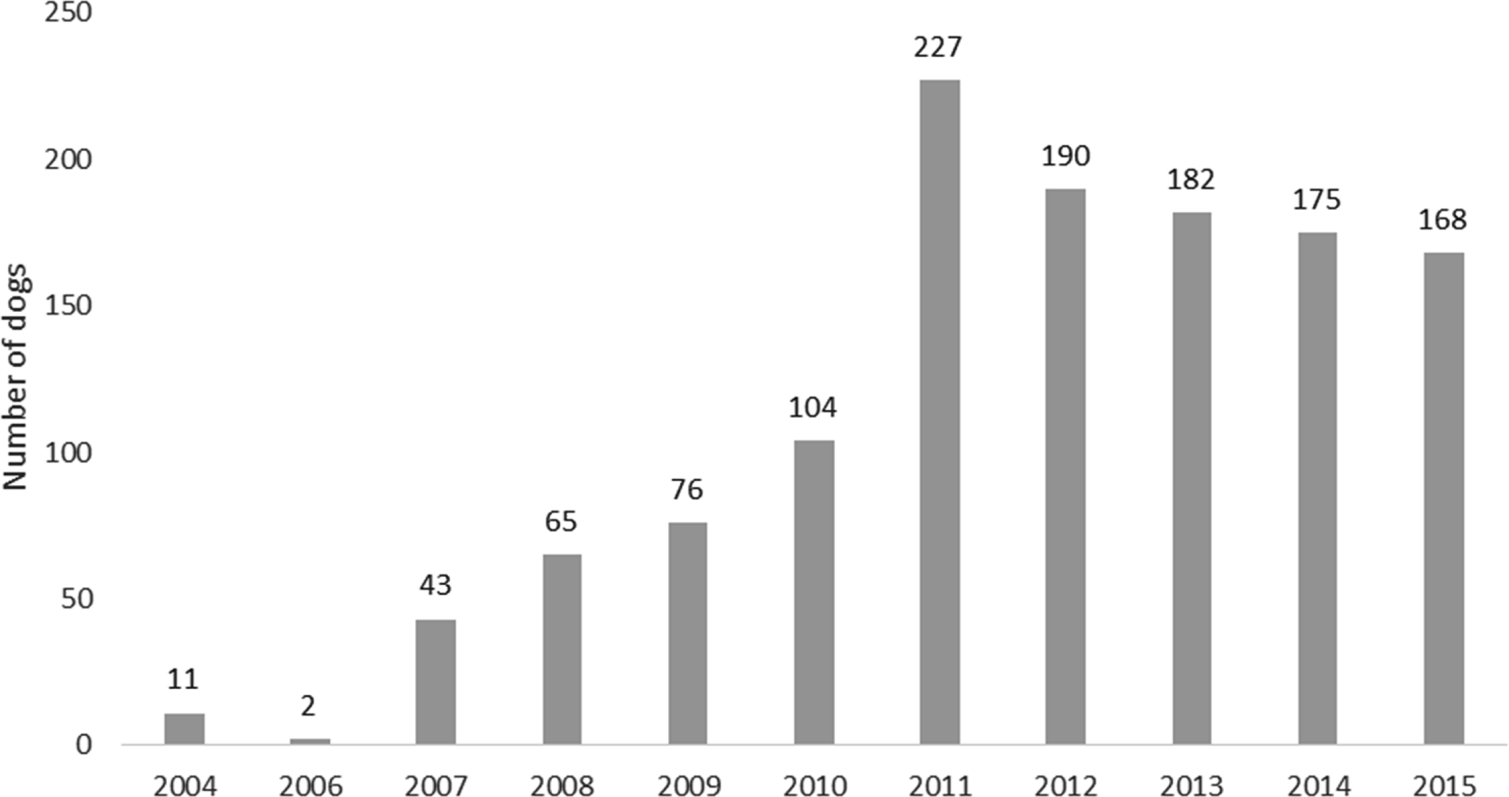
Number of total MRI scans per year of Cavalier King Charles spaniels collected in the Netherlands and Belgium for screening of syringomyelia and Chiari-like malformation.

During the time frame of January 2004 and December 2015, a total of 1249 MRI scans were made. Compared with 2007 the number of scans made gradually increased to approximately 170 scans a year in 2015 (see Fig 1).

A total of 1108 out of 1249 MRI scans were produced in Best, 39 in Meppel, 13 in Amsterdam, 35 in Dordrecht, 12 in Drachten, 22 in Herentals, and a total of 20 in Utrecht. Although all scans were made from pedigree dogs intended for breeding, from a number of dogs the gender (n = 56), fur color (n = 104) or clinical status (n = 400) could not be retrieved. From 217 dogs, multiple scans were made, 205 dogs were scanned twice, and twelve dogs three times. Only the first and last scan was used for evaluation. As such, the dataset contains 1020 unique dogs (696 females, 268 males and 56 of unknown gender). According to the written submission forms, 608 of these dogs did not suffer from clinical signs suggestive for SM and/or CM whereas 11 dogs (7 females and 4 males) showed suggestive signs for CM and/or SM. One female dog that was scanned twice did not have any clinical signs at the first scan, but at the moment of the second scan clinical signs suggestive for CM and/or SM were observed. For 400 dogs the status was unknown. There was a small but statistically significant correlation of 0.2 for the presence of clinical signs and a higher CM grade (p = 0.000), as well as a small correlation of 0.12 for an increase of central canal dilation and the presence of clinical signs (P = 0.000).

**Fig 2:**
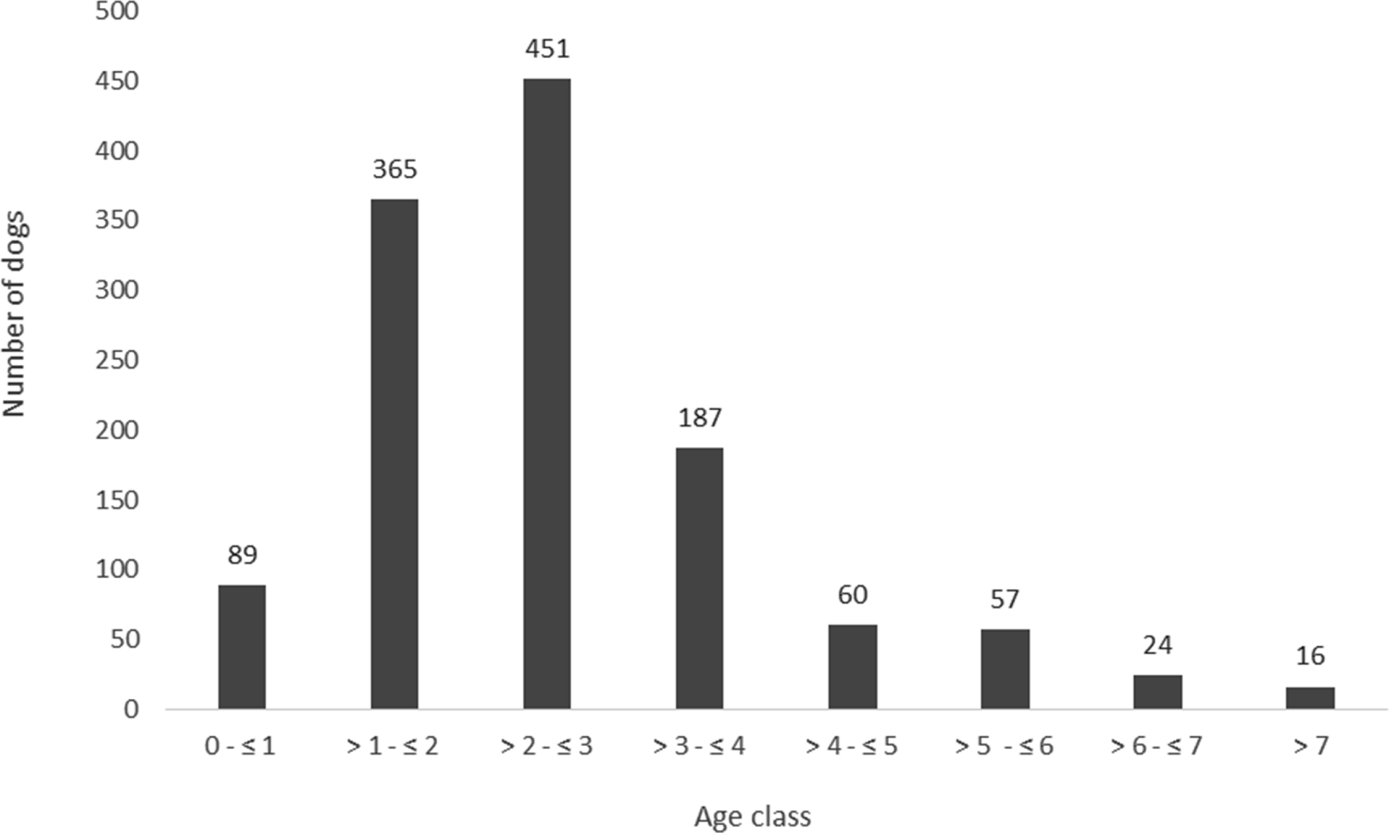
Overview of the number of dogs that were screened per age class.

Of the 1020 unique dogs in this study, 444 dogs were Blenheim’s, 231 Tricolors, 129 Black and Tan’s, 112 Ruby’s. Of 104 dogs the fur color could not be retrieved. There was no significant statistical difference for the proportions of fur colors over the years (p = 0.78). Hence fur colors were equally divided throughout the years. The majority of scans were produced from dogs between 1 to 3 years of age (n = 809). A total number of 351 scans were produced from dogs older than 3 years of age and 89 of dogs between 0 and 1 year of age (Fig 2).

Only 523 MRI scans did not show any microchip artifacts. In 630 MRI scans microchip artifacts were present but did not interfere with the evaluation, in 90 MRI scans artifacts did interfere but the evaluation was still possible. In 6 scans, the microchip artifact prevented a proper evaluation of the scan. Hence only 1243 MRI scans remained for further evaluation.

### CM status

Using the BVA/KC scheme for CM, 18 out of 1243 dogs were scored as CM1, 1225 out of 1243 dogs were scored as CM2. Using the adapted scheme 18 dogs were graded as CM1, 382 as CM2, 818 as CM3 and 25 out of 1243 scans as CM4 (see Table 1). There was no significant difference in CM classification if the dogs were divided into the three age classes (t-test, p-value = 0.24). Hence the CM grading did not worsen over time.

**Table 1:** Overview of the CM status of the different scans according to the BVA/KC scheme and the adapted scheme, and the division over the different age classes. Age class an equals dogs older than 5 years, b between 3 and 5 years and c younger than 3 years.

BVA/KC		Adapted scheme	
**CM grade 0**	0	**CM grade 0**	0
**CM grade 1**	18	**CM grade 1**	18
Age a	0		0
Age b	3		3
Age c	15		15
**CM grade 2**	1225	**CM grade 2**	382
Age a	102	Age a	36
Age b	251	Age b	79
Age c	869	Age c	267
		**CM grade 3**	818
		Age a	65
		Age b	168
		Age c	585
		**CM grade 4**	25
		Age a	1
		Age b	4
		Age c	20

### SM status

A total of 760 dogs were graded as SM0, 288 as SM1 and 195 out of 1243 as SM2 (see Table 2). Divided for the three age groups there was a clear significant statistical difference between the age classes (t-test, p < 0.001). The number of dogs that were graded with SM1 and SM2 increased with age, clearly suggesting that the disorder increases over time.

**Table 2:** Overview of the SM status of the different scans according to the BVA/KC scheme, and the division over the different age classes. Age class a equals dogs older than 5 years, b between 3 and 5 years and c younger than 3 years.

	BVA/KC
**SM grade 0**	760
Age a	41
Age b	119
Age c	600
**SM grade 1**	288
Age a	29
Age b	73
Age c	186
**SM grade 2**	195
Age a	33
Age b	64
Age c	98

### Central canal dilation (CCD) over time

Divided into age groups from <1 years up to 7 years and more, there was again a clear increase of CCD over time. The CCD increases over time (see Table 3) and this increase was statistically significant (ANOVA; p = 0.004).

**Table 3:** Average CCD ± SD and the number of dogs scanned per age category for the dogs that were able to be evaluated (dogs without chip artifact).

Age group	Number of dogs	Average CCD ± SD (mm)
**0–≤ 1**	89	0.21 ± 0.57
**> 1–≤ 2**	364	0.41 ± 0.94
**> 2–≤ 3**	447	0.82 ± 1.31
**> 3–≤ 4**	187	1.15 ± 1.53
**> 4–≤ 5**	60	1.37 ± 1.62
**> 5–≤ 6**	56	1.79 ± 2.18
**> 6–≤ 7**	24	1.46 ± 1.62
**> 7**	16	1.38 ± 1.45

### Central canal dilation (CCD) in dogs scanned multiple times

Out of the 217 dogs that were scanned multiple times, 205 were scanned twice, and 12 were scanned 3 times. The average age at the first scan for these dogs was 1.5 ± 0.6 years. A total of 174 dogs had no CCD at the time of their first scan, and 43 dogs did. The average age of this group of 43 dogs was 1.7 ± 0.6 years and was not significantly different from the dogs that had no CCD. The average CCD was 1.3 ± 1.1 mm. At the last scan (either the second if only two scans were made or third if three were made) the average age for this group of 43 dogs was 3.1 ± 0.9 years and the CCD at that time was 2.1 ± 1.1 mm. Within the complete group of 217 dogs, 132 dogs (97 females, 33 males) had no CCD at their first—and final scan. Eighty-five (62 females, 23 males) out of these 217 dogs however, of which 43 already had some degree of CCD, had an (increase) of their CCD (see S1 Fig). There was no significant difference in the age or sex of these two groups (dogs having no CCD at first and final scan compared to the other dogs).

### SM and CM correlation

A small but statistical significant correlation of 0.184 could be noted for CM grading and CCD (p = 0.000).

### Middle ear effusion

The presence of middle ear effusion was recorded in all dogs. For the group of dogs younger than 3 years of age (809 out of 1020 dogs), 19% presented middle ear effusion in the left bullae and 22% in their right bullae. This percentage had risen to 32% (left bullae) and 38% (right bullae) for dogs older than three years of age (211 out of 1020 dogs). Interestingly the same increase was noted for the dogs that were scanned multiple times. The number of “middle-ear effusion free” dogs decreased from 82% to 72% (left middle ear) and 79% to 70% (right middle ear) respectively. There was no correlation for the CM grading and the presence of either left (p = 0.47) or right middle ear effusion (p = 0.22) or for dogs that had in both middle ears middle ear effusion (p = 0.96). However a small statistical significant correlation of 0.1 was observed for CCD and both left and right middle ear effusion (p = 0.000).

### Genetic parameters for the SM and CM phenotypes

Of all 1020 dogs available in the database, only 906 could be linked to pedigree dogs present in the database of the Cavaliers for Life foundation. So genetic analysis was restricted to these 906 dogs. Out of these 906 dogs, 500 of them had their mothers screened, whereas 302 their fathers.

### Exploratory analysis of variance

The factors that had a significant influence on grade of SM (both CCD and the BVA/KC) were the age of the dog at screening (p-value = 0.005) as well as the effect “veterinary clinic taking the MRI scan*year of the scan (VCY)” (p-value = 0.003). Other factors such as sex, owner, and color of the dog were not significant. For CM, only the VCY factor had a significant effect (p-value = 0.019).

### Genetic parameters and heritabilities for SM and CM

Variance components and genetic parameters for both models of SM are shown in Table 4. The model with age and VCY had a lower AIC value for both phenotypes compared to the model with only age, which indicates that the former is a better fitting model. For this model, a moderate heritability (SE) for the CCD was found at 0.301. VCY accounted for a large proportion of the variance (3.175). The total phenotypic variance was 5.128. When comparing to the analysis based on SM according to the scoring system of the BVA/KC, heritability was lower at 0.127, VCY variance was 2.6335 and total phenotypic variance 3.196. The model with only age resulted in a higher heritability, and this for both phenotypes (0.75 and 0.79 respectively), but also respective AIC values were much higher (3180 and 2308 compared to 3078 and 2015).

**Table 4:** Variance components and heritabilities for the width of the syrinx (CDD) and SM according to the BVA/KC scheme. Comparison of a model with only an age effect and a model with both age and veterinary clinic*year effect (VCY). AIC = Akaike Information Criterion.

	Model: Age		Model: Age + VCY	
	CCD (mm)	BVA/KC scheme	CCD (mm)	BVA/KC scheme
**AIC**	3180	2308	3078	2015
$\sigma_{p}^{2}$	2.341	0.909	5.128	3.196
$\sigma_{a}^{2}$	1.758	0.719	1.541	0.406
$\sigma_{vet*yr}^{2}$	—	—	3.175	2.634
$\sigma_{e}^{2}$	0.583	0.190	0.412	0.156
***H***^***2***^	0.751	0.791	0.301	0.127

Therefore, further analysis for SM considered the results of the models with both age and VCY.

Variance components for CM (BVA/KC score and new scheme) are represented in Table 5. The variance due to VCY effect accounted for a large proportion of the variance (3.647 and 6.626). Heritabilities for this population were very low at 0.022 for the BVA/KC scoring method, and 0.031 for the new scheme, but in the same range for both schemes.

**Table 5:** Variance components and heritabilities for CM according to the BVA/KC classification, and the newly developed scheme, and this for a model with only a veterinary clinic*year effect (VCY).

	Model: Age + VCY	
	BVA/KC scheme	New scheme
$\sigma_{p}^{2}$	3.730	7.022
$\sigma_{a}^{2}$	0.082	0.218
$\sigma_{vet*yr}^{2}$	3.647	6.626
$\sigma_{e}^{2}$	0.001	0178
***h***^***2***^	0.022	0.031

Genetic correlations between all CM and SM measures are presented in Table 6. The genetic correlation between both SM measures (BVA/KC and CCD) and both CM measures (BVA/KC and new scheme) were found to be very high, 0.97 and 0.83 respectively. Correlations between the SM and CM evaluation were rather low but positive, ranging between 0.16 and 0.32.

**Table 6:** Genetic correlations between SM and CM measures.

	SM- BVA/KC	SM—CCD	CM–BVA/KC	CM–new
**SM- BVA/KC**	-			
**SM—CCD**	0.97	-		
**CM–BVA/KC**	0.23	0.33	-	
**CM–new**	0.16	0.25	0.83	-

**Fig 3:**
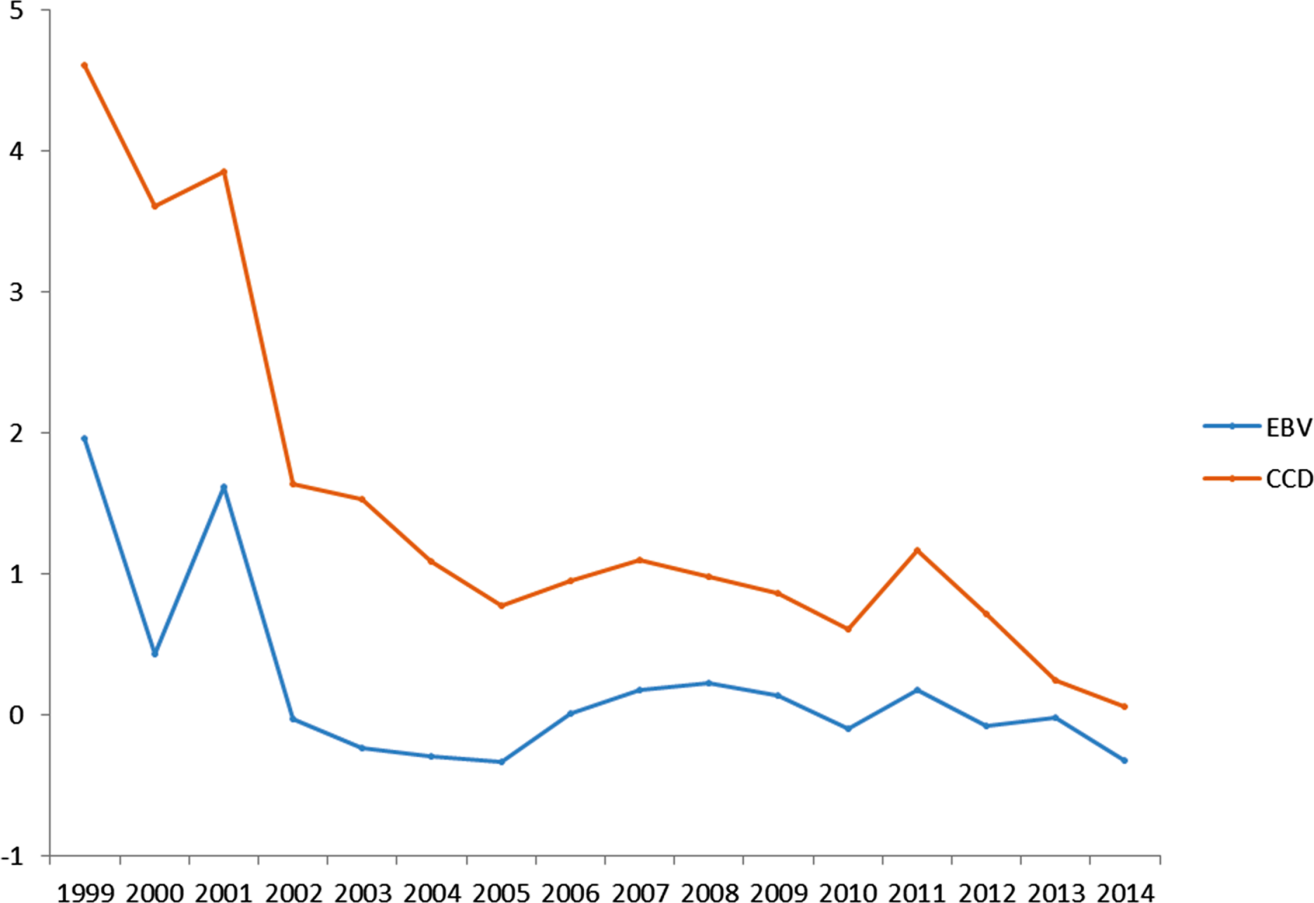
Average estimated breeding values according to the classical evaluation method (BVA/KC scheme) (EBV) and according to the newly developed method (CCD) (Mms) by birth year.

Fig 3 shows the distribution of the mean EBV for the BVA/KC scheme and CCD per birth year. We notice a gradual decrease in the average EBVs over the years, indicating some genetic progress against SM. It is noteworthy that this figure only includes the dogs that had own records.

**Fig 4:**
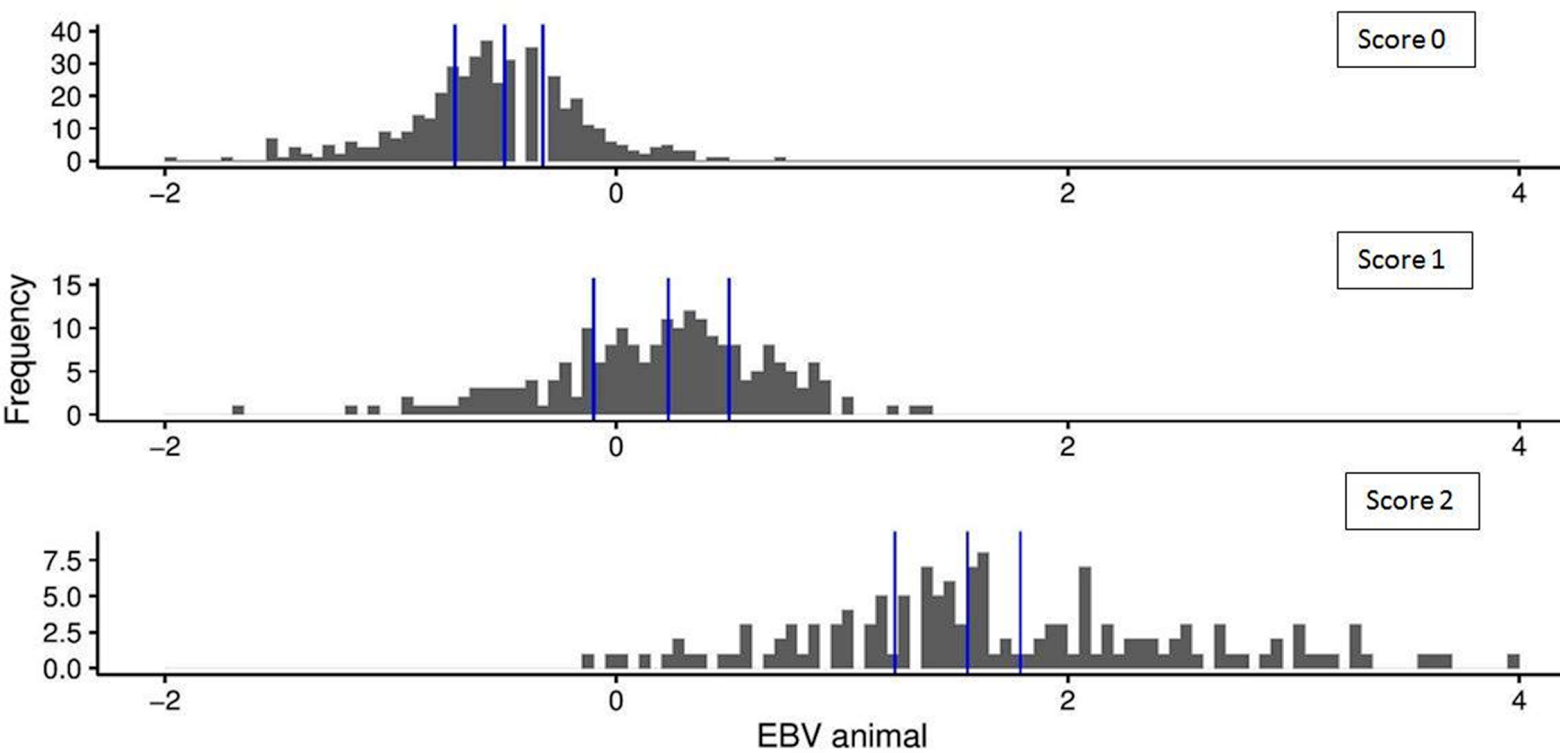
Distribution of EBVs over the phenotypic scores. The different graphs represent the BVA/KC score 0, 1 or 2 respectively. Blue lines divide the distribution into quartiles. Only dogs with their own phenotypic score are represented.

The distribution of the EBVs of the dogs over the three different phenotypic scores for SM (0, 1 or 2) is shown in Fig 4. The overlapping of these distributions reveals that a large range of EBVs can occur within one phenotype. Also here, only dogs with their own phenotypic score are represented.

## Discussion

Despite the fact that all scans were made from pedigree CKCS, some data concerning color, gender or actual clinical status, were irretrievable. Various reasons could account for this. First of all most MRI scans were retrieved from the MRI-center long after the actual scanning date. Although in most cases the MRI scan contained pedigree numbers or microchip numbers, especially the older scans lacked information on color, gender and clinical signs. As of 2011, the Dutch breeders are obliged to provide the evaluation team all necessary data including information on clinical signs. Breeders are not allowed to use a dog if it shows any clinical signs suggestive for CM or SM. Analysis of the data on color or gender, just as in previous studies, did not reveal any relation with CM, SM or middle ear effusion. Interestingly CM was found to be present in almost all dogs in the population. None of the dogs had a grade 0, whereas only 18 (1.4%) had a grade 1. This condition has already been reported in a number of other breeds, mostly toy breeds, such as among others Affenpinschers, Griffon Bruxellois and Maltese (4,20). In the Griffon Bruxellois for example, prevalence can reach up to 65%, depending on the investigated population [20,21]. Other studies on CKCS also found very high prevalence for CM, up to 95% [1].

The majority of the dogs scanned were below three years of age. In this group the prevalence for SM1 was 21% and for SM2 11%. However, the percentage of SM1 and SM2 increased significantly the older the dog became. The prevalence rose to 28% for SM1 and 25% for SM2 in the age group 3 to 5 years, and to 28% (SM1) and 32% (SM2) for dogs older than 5 years of age. Similar results were found earlier [1,22]. Parker and others [22] found a prevalence up to 70% but the difference might be caused by the fact that breeders do not voluntarily scan their dog repeatedly if the dog was found to suffer from SM2 at a younger age. For the same reason it is difficult to estimate the actual SM status of the complete Dutch population at older age. An older dog, not intended for breeding, is only scanned if there are clinical signs. And in this study we only selected dogs intended for breeding and these are not allowed to have clinical signs. The fact that so many dogs in this study had SM without clinical signs has been reported earlier. Rusbridge and others [8] demonstrated that only up to about 35% of dogs having SM actually show clinical signs. When subdividing the dogs into various age groups and scoring the CDD in actual millimeters, the age effect is clearly seen. Younger dogs received lower scores than older dogs, and this was found to be significant in this study. This has also been confirmed in the studies of Parker and others [22] and Driver and others [5], who found that the maximum syrinx width, height of the foramen magnum, length of the cerebellar herniation and the caudal cranial fossa volume increased over time. This also stresses the importance of screening at appropriate ages, as the dog develops SM over time. This implies that dogs might have been bred before SM was diagnosed. In our data, about 48% of the dogs were under 3 years when screened (score SM0, age c). Given the natural process of SM, it is likely that a number of these dogs ultimately become SM-affected (SM2). Of the dogs that were scanned multiple time only 132 out of 217 dogs remained SM0 in time. However 85 dogs, of which 43 already had some degree of CCD, worsened over time. And strikingly we observed that dogs without any CCD at the first scan worsened dramatically over time, while other dogs with minimal CCD at first only developed slightly higher scores at the second or third scan. Knowler and others [23] demonstrated a relationship with SM status of the ancestor. An SM free dog with SM free parents is more likely to have SM free offspring compared to an SM free dog with parents that have SM. For this reason the use of pedigree data to estimate breeding values (EBVs) is more efficient than phenotypic selection to reduce the prevalence of SM.

In this study, the prevalence of middle ear effusion was assessed and estimated at 19%–21% for dogs younger than 3 years, and 32%–38% for dogs older than 3. The condition has been diagnosed with various clinical signs, and it has been associated with brachycephalic conformation [24]. It is currently under debate whether the behaviors that are associated with pain due to SM in the CKCS are related to middle ear effusion. In other CKCS populations, estimates of the presence of middle ear effusion reach about 32%–70% [24,25]. There was no correlation, in this study, for CM and the presence of middle ear effusion, which seems to suggest that middle ear effusion is more associated with a brachycephalic conformation rather than the actual CM grading. This is remarkable as CM is clearly associated with a brachycephalic conformation [3,7]. Remarkably, a small but statistically significant correlation was found for CDD and middle ear effusion. If this observation is correct it merits further research, as it has not been investigated before. Furthermore it must be noted that in a CKCS, it might be difficult to notice clear signs that could be caused by the middle ear effusion as most of them suffer from CM as well.

In this study the presence of microchip artifacts was also studied. Given the fact that up to 726 out of 1253 scans showed artifacts of which 90 made the evaluation difficult and in six impossible it is suggested to place the microchips in breeds at risk of developing SM not in the neck region. Ideally they are placed, for instance, behind the shoulder blades.

The current BVA/KC scheme scores SM at 3 levels and further subdivides the grade into three age categories based on the CCD. However using the actual width of CCD yields a higher heritability estimate. In this cohort heritability for SM using the CCD phenotype (0.30) is higher compared to a model using the BVA/KC scheme evaluation method (0.13). This increases the potential of selective breeding in order to reduce the prevalence against SM. Both models included an effect of the veterinarian clinic taking the MRI scan, as well as the age of the dogs at screening. By including age in the statistical model, there is no need for breeding rules based on “age at scanning”, since there is an automatic correction for age. Therefore, we propose to change the current BVA/KC scheme and measure CCD in mm’s. Up until now, only two other studies looked into the heritability of SM in the CKCS. Lewis et al. (2010) found a moderate heritability when looking at presence of SM (affected/clear phenotype) in 384 Kennel Club registered CKCS in the period of 1998 to 2009 [14]. They estimated the heritability of SM at 0.37 (± 0.15), with significant effects of year of birth and age at scanning. Thøfner and others [15] on the other hand investigated only symptomatic CKCS and found a much higher heritability of 0.81. The veterinary clinic year effect was significant and produced a large proportion of the variance on the grade of SM as well as CM. As all MRI scans were made using the same protocol and produced comparable studies it must be a true veterinary clinic year effect. A very large proportion of the breeders preferred to go to one specific clinic (Best), and this may accidently have introduced some bias as it seem logical that this clinic and its employees are most likely better trained in the positioning of the dogs to produce the best quality of MRI scans. One could raise the question whether it might be prudent to limit this type of screening to those clinics that have a sufficient number of cases to improve the quality of the MRI and hence the evaluation of the MRI scan.

The high genetic correlation between the BVA/KC scoring method and the measurement of CCD of 0.97 is not surprising, as the BVA/KC scoring method is based on the measurement of the CCD. However, by classifying the dogs, detailed information about the severity of the CCD is lost.

Also for CM, the heritability was compared between the BVA/KC scheme and a newly developed method. They were both very low (0.02 and 0.03) as to be expected as the heritability estimates were computed as a proportion of the phenotypic variance. And this was small in this population. But it is interesting to have a more detailed scoring of CM. Due to the omnipresence of CM in the breed, it will be challenging to make fast progress due to the low heritability calculated. Faster progress can be obtained with crossbreeding. The effect of crossbreeding was demonstrated in the Griffon Bruxellois, where an outcross with a CM-free Australian terrier resulted in a F1 generation with some CM-free crosses. By carefully selecting appropriate conformation, it is expected that CM grade will be reduced, while crosses regain close resemblance to the actual Griffon Bruxellois breed [26].

The high genetic correlation of 0.83 between both methods of scoring CM is not surprising, given the division of the most severe class in 3 subcategories. Furthermore, the genetic correlation between CM and SM was rather low but positive at 0.16–0.32, which indicates that by selecting against SM, CM will slightly ameliorate as well.

As mentioned before, selection based on EBVs could be more effective than selection on the phenotype alone. EBVs are more accurate than phenotypic selection since they take into account the genetic contribution of all relatives (parents and offspring), and the information of the individual itself. It has already been demonstrated in other complex diseases such as canine hip dysplasia that EBVs will lead to an improvement in the response of selection [11,14,27–29].

The advantage of using EBVs instead of the phenotypes for SM was also demonstrated in Fig 4, where dogs having the same BVA/KC score can have a wide range of EBVs. Using EBVs will give a more precise selection criterion. However, the use of EBVs as a selection criterion implies that screening is continued. The accuracy of the EBVs will increase as more information will become available (such as own measurements or measurements of relatives). Another advantage of EBVs is the ability to obtain EBVs for dogs, at birth, without own phenotypical records. However as heritability is moderate it must be stressed that the dogs own phenotype is very important and scanning of breeding dogs continues to be needed. Furthermore the reliability of the EBV will improve greatly if as many dogs as possible will be scanned.

Given the moderate genetic correlations with CM (Table 6), selection for SM is expected to aid the improvement of CM as well.

In conclusion the results underline that it is necessary to continue the screening for both CM and SM for the entire CKCS population. Moreover, to avoid artefacts and achieve more “usable” scans, microchips should not be located in the neck region.

Reduction of CM by selection at this stage however may be very hard to achieve given the low heritability of CM-scores based on MRI-scans. However, the positive genetic correlation between SM and CM will aid the selection against CM. The current application of breeding rules (against SM) results in only little progress over time. The moderate heritability suggests that selection using EBVs will be more effective. Hence scanning data should be converted to EBVs and used in a selection program against SM and CM.

## Supporting information

S1 Fig CCD scans. Representation of all dogs with the length of the CCD during their first scan (blue) and their second scan (orange).
(TIFF)

S1 Table MRI data. Excel file with all, anonymized, analyzed MRI scan.
(XLSX)

## References

[1] Rusbridge C. Chiari-like malformation and Syringomyelia in the Cavalier King Charles Spaniel. PhD Thesis. University of Utrecht; 2007. 10.1111/j.1532-950X.2007.00285.x 17614920

[2] Driver CJ, Volk HA, Rusbridge C, Van Ham LM. An update on the pathogenesis of syringomyelia secondary to Chiari-like malformations in dogs. Vet J [Internet]. 2013;198(3):551–9. Available from: http://dx.doi.org/10.1016/j.tvjl.2013.07.014 23938004 10.1016/j.tvjl.2013.07.014

[3] Shaw TA, McGonnell IM, Driver CJ, Rusbridge C, Volk HA. Increase in cerebellar volume in Cavalier King Charles Spaniels with Chiari-like malformation and its role in the development of syringomyelia. PLoS One. 2012;7(4). 10.1371/journal.pone.0033660 PMC3323625 22506005

[4] Marino DJ, Loughin C a, Dewey CW, Marino LJ, Sackman JJ, Lesser ML, et al Morphometric features of craniocervical junction region in dogs with suspected Chiari-like malformation determined by combined use of magnetic resonance imaging and computed tomography. Am J Vet Res. 2012;73(1):105–11. doi: 10.2460/ajvr.73.1.105 22204295 10.2460/ajvr.73.1.105

[5] Driver CJ, De Risio L, Hamilton S, Rusbridge C, Dennis R, McGonnell IM, et al Changes over time in craniocerebral morphology and syringomyelia in cavalier King Charles spaniels with Chiari-like malformation. BMC Vet Res [Internet]. 2012;8:215 Available from: http://www.ncbi.nlm.nih.gov/pubmed/23136935%5Cnhttp://www.biomedcentral.com/content/pdf/1746-6148-8-215.pdf doi: 10.1186/1746-6148-8-215 23136935 10.1186/1746-6148-8-215 PMC3514376

[6] Cross HR, Cappello R, Rusbridge C. Comparison of cerebral cranium volumes between cavalier king charles spaniels with chiari-like malformation, small breed dogs and labradors. J Small Anim Pract. 2009;50(8):399–405. doi: 10.1111/j.1748-5827.2009.00799.x 19689667 10.1111/j.1748-5827.2009.00799.x

[7] Knowler SP, McFadyen AK, Freeman C, Kent M, Platt SR, Kibar Z, et al Quantitative analysis of Chiari-like malformation and syringomyelia in the Griffon Bruxellois dog. PLoS One. 2014;9(2). 10.1371/journal.pone.0088120 PMC3922758 24533070

[8] Rusbridge C, Carruthers H, Dubé MP, Holmes M, Jeffery ND. Syringomyelia in cavalier King Charles spaniels: The relationship between syrinx dimensions and pain. J Small Anim Pract. 2007;48(8):432–6. doi: 10.1111/j.1748-5827.2007.00344.x 17608656 10.1111/j.1748-5827.2007.00344.x

[9] Falconer D, Mackay T. Introduction to quantitative genetics 4th ed. Longman; 1996. 457 p.

[10] Miglior F, Muir BL, Van Doormaal BJ. Selection indices in Holstein cattle of various countries. J Dairy Sci [Internet]. 2005;88(3):1255–63. Available from: http://dx.doi.org/10.3168/jds.S0022-0302(05)72792-2 15738259 10.3168/jds.S0022-0302(05)72792-2

[11] Malm S, Fikse WF, Danell B, Strandberg E. Genetic variation and genetic trends in hip and elbow dysplasia in Swedish Rottweiler and Bernese Mountain Dog. J Anim Breed Genet. 2008 12;125:403–12. doi: 10.1111/j.1439-0388.2008.00725.x 19134076 10.1111/j.1439-0388.2008.00725.x

[12] Silvestre a M, Ginja MMD, Ferreira a J a, Colaço J. Comparison of estimates of hip dysplasia genetic parameters in Estrela Mountain Dog using linear and threshold models. J Anim Sci. 2007 8;85:1880–4. doi: 10.2527/jas.2007-0166 17468417 10.2527/jas.2007-0166

[13] Lewis T, Blott SC, Woolliams JA. Comparative analyses of genetic trends and prospects for selection against hip and elbow dysplasia in 15 UK dog breeds. BMC Genet [Internet]. 2013;14(1):16. Available from: BMC Genetics 23452300 10.1186/1471-2156-14-16 PMC3599011

[14] Lewis T, Blott SC, Woolliams J a. Genetic evaluation of hip score in UK Labrador Retrievers. PLoS One. 2010 1;5(10):e12797 doi: 10.1371/journal.pone.0012797 21042573 10.1371/journal.pone.0012797 PMC2962628

[15] Thøfner MS, Stougaard CL, Westrup U, Madry AA, Knudsen CS, Berg H, et al Prevalence and Heritability of Symptomatic Syringomyelia in Cavalier King Charles Spaniels and Long-term Outcome in Symptomatic and Asymptomatic Littermates. J Vet Intern Med. 2015;29(1):243–50. doi: 10.1111/jvim.12475 25308931 10.1111/jvim.12475 PMC4858089

[16] Lewis T, Rusbridge C, Knowler P, Blott S, Woolliams J a. Heritability of syringomyelia in Cavalier King Charles spaniels. Vet J. 2010 3;183:345–7. doi: 10.1016/j.tvjl.2009.10.022 19914109 10.1016/j.tvjl.2009.10.022

[17] Rosset A, Spadola L, Ratib O. OsiriX: An open-source software for navigating in multidimensional DICOM images. J Digit Imaging. 2004;17(3):205–16. doi: 10.1007/s10278-004-1014-6 15534753 10.1007/s10278-004-1014-6 PMC3046608

[18] Akaike H. A new look at the statistical model identification. IEEE Trans Autom Control. 1974;19(6):716–23.

[19] Misztal I, Tsuruta S, Strabel T, Auvray B, Druet T, Lee DH. BLUPF90 and related programs (BGF90). In: 7th World Congress on Genetics Applied to Livestock Production, Montpelier, 19–23 August. Montpellier; 2002.

[20] Freeman AC, Platt SR, Kent M, Huguet E, Rusbridge C, Holmes S. Chiari-Like Malformation and Syringomyelia in American Brussels Griffon Dogs. J Vet Intern Med. 2014;28(5):1551–9. doi: 10.1111/jvim.12421 25145262 10.1111/jvim.12421 PMC4895564

[21] Rusbridge C, Knowler SP, Pieterse L, McFadyen a K. Chiari-like malformation in the Griffon Bruxellois. J Small Anim Pract. 2009 8;50:386–93. doi: 10.1111/j.1748-5827.2009.00744.x 19689665 10.1111/j.1748-5827.2009.00744.x

[22] Parker JE, Knowler SP, Rusbridge C, Noorman E, Jeffery ND. Prevalence of asymptomatic syringomyelia in Cavalier King Charles spaniels. Vet Rec. 2011;168:667 doi: 10.1136/vr.d1726 21672954 10.1136/vr.d1726

[23] Knowler SP, McFadyen AK, Rusbridge C. Effectiveness of breeding guidelines for reducing the prevalence of syringomyelia. Vet Rec [Internet]. 2011;169(26):681 Available from: http://www.ncbi.nlm.nih.gov/pubmed/21998144 doi: 10.1136/vr.100062 21998144 10.1136/vr.100062

[24] Hayes GM, Friend EJ, Jeffery ND. Relationship between pharyngeal conformation and otitis media with effusion in Cavalier King Charles spaniels. Vet Rec [Internet]. 2010;167(2):55–8. Available from: http://veterinaryrecord.bmj.com/content/167/2/55.abstract doi: 10.1136/vr.b4886 20622204 10.1136/vr.b4886

[25] Stern-Bertholtz W, Sjöström L, Håkanson NW. Primary secretory otitis media in the Cavalier King Charles spaniel: a review of 61 cases. J Small Anim Pract. 2003 6;44(6):253–6. 12831101 10.1111/j.1748-5827.2003.tb00151.x

[26] Knowler SP, v/d Berg H, McFadyen A, La Ragione RM, Rusbridge C. Inheritance of Chiari-Like Malformation: Can a Mixed Breeding Reduce the Risk of Syringomyelia? PLoS One [Internet]. 2016;11(3):e0151280 Available from: http://dx.plos.org/10.1371/journal.pone.0151280 doi: 10.1371/journal.pone.0151280 27008271 10.1371/journal.pone.0151280 PMC4805231

[27] Hou Y, Wang Y, Lust G, Zhu L, Zhang Z, Todhunter RJ. Retrospective analysis for genetic improvement of hip joints of cohort labrador retrievers in the United States: 1970–2007. PLoS One. 2010 1;5(2):e9410 doi: 10.1371/journal.pone.0009410 20195372 10.1371/journal.pone.0009410 PMC2827553

[28] Lewis T, Woolliams JA, Blott SC. Optimisation of breeding strategies to reduce the prevalence of inherited disease in pedigree dogs. Anim Welf. 2010;19:93–8.

[29] Lewis T, Ilska J, Blott S, Woolliams J. Genetic evaluation of elbow scores and the relationship with hip scores in UK Labrador retrievers. Vet J. 2011 8;189(2):227–33. doi: 10.1016/j.tvjl.2011.06.024 21737324 10.1016/j.tvjl.2011.06.024

