# Supplementary figures and images for: Twelve years of chiari-like malformation and syringomyelia scanning in Cavalier King Charles Spaniels in the Netherlands: Towards a more precise phenotype
Source: PLoS One. 2017 Sep 21;12(9):e0184893. doi: 10.1371/journal.pone.0184893 (PMC5608246; doi:10.1371/journal.pone.0184893)

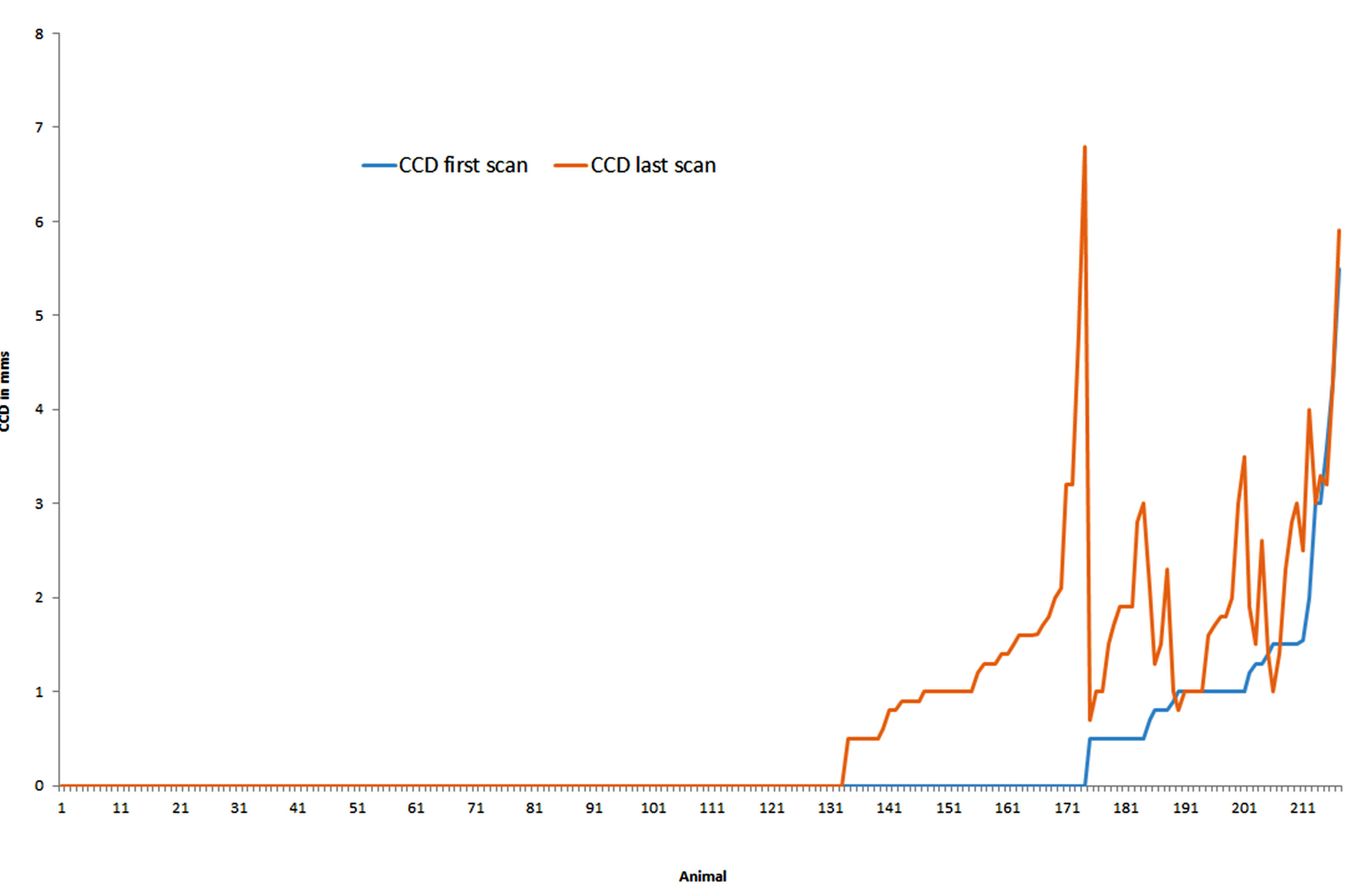

Supplement: S1 Fig — Representation of all dogs with the length of the CCD during their first scan (blue) and their second scan (orange). (TIFF) [file pone.0184893.s001.tiff]
